# Anti-inflammatory dietary interventions in inflammatory bowel disease: current insights and future perspectives

**DOI:** 10.3389/fnut.2026.1817465

**Published:** 2026-05-11

**Authors:** Mingmin Li, Chuan Jiang, Benjun Wang, Zhongan Guan, Li Li

**Affiliations:** 1 Qilu Hospital of Shandong University, Jinan, China; 2 First Clinical Medical School, Shandong University of Traditional Chinese Medicine, Jinan, China; 3 Department of Anorectal Surgery, Affiliated Hospital of Shandong University of Traditional Chinese Medicine, Jinan, China; 4 Department of Anorectal Surgery, Qingdao Hiser Hospital Affiliated of Qingdao University, Qingdao, China

**Keywords:** anti-inflammatory dietary, Crohn's disease, inflammation, inflammatory bowel disease, Mediterranean diet (MD), ulcerative colitis

## Abstract

Inflammatory bowel disease (IBD), encompassing Crohn's disease (CD) and ulcerative colitis (UC), arises from multifactorial interactions involving genetics, gut microbiota dysbiosis, immune dysregulation, and environmental triggers, with diet playing a pivotal role. Anti-inflammatory dietary patterns have emerged as adjunctive therapies to pharmacological treatments, potentially inducing and maintaining remission by modulating microbiota and inflammation. This perspective reviews the mechanistic basis for dietary interventions, evaluates key strategies such as the Mediterranean diet (MD), Specific Carbohydrate Diet (SCD), low-FODMAP diet, IBD-Anti-Inflammatory Diet (IBD-AID), and Groningen Anti-Inflammatory Diet (GrAID), and discusses clinical evidence, challenges, and implications. While promising, evidence is constrained by heterogeneous studies and small cohorts. Future research should prioritize large-scale randomized controlled trials (RCTs) with multi-omics integration for personalized nutrition in IBD management.

## Introduction

Inflammatory bowel disease (IBD) represents a group of chronic, relapsing inflammatory disorders of the gastrointestinal tract, primarily comprising Crohn's disease (CD) and ulcerative colitis (UC). UC is characterized by continuous mucosal inflammation starting in the rectum and extending proximally, while CD can affect any segment of the gastrointestinal tract discontinuously, often with transmural involvement (1). The global incidence of IBD is rising, particularly in industrialized regions, with environmental factors, including diet, implicated in disease onset and progression (2). Recent epidemiological data indicate that the prevalence of IBD exceeds 0.3% in North America and Europe, with emerging increases in Asia and Latin America, underscoring the influence of Westernization on disease patterns (3).

Diet influences IBD pathogenesis through interactions with the gut microbiota, epithelial barrier integrity, and immune responses. Western diets high in ultra-processed foods, refined sugars, saturated fats, and additives promote dysbiosis, favoring pro-inflammatory bacteria and reducing short-chain fatty acid (SCFA) producers like *Faecalibacterium prausnitzii* (4). This dysbiosis contributes to a leaky gut barrier, allowing luminal antigens to trigger aberrant immune responses, including Th1/Th17 polarization in CD and Th2 in UC (5). Conversely, anti-inflammatory diets rich in fiber, omega-3 fatty acids, polyphenols, and prebiotics enhance microbial diversity, SCFA production, and mucosal homeostasis (6). For instance, dietary fiber fermentation yields butyrate, which fuels colonocytes and suppresses pro-inflammatory cytokines (7).

Over the past decade, dietary interventions have gained recognition as safe, patient-centered complements to biologics and immunosuppressants, potentially reducing flares and improving quality of life (8). Patient surveys reveal that up to 66.8% of IBD individuals modify their diet to manage symptoms, yet evidence-based guidance remains limited (9). The latest Dietary Guidelines for Americans (2025–2030) (10) strongly endorse healthy dietary patterns that align closely with anti-inflammatory principles, emphasizing abundant fruits, vegetables, whole grains, legumes, nuts, healthy fats (e.g., from olive oil and fish), and limited added sugars, saturated fats, and sodium—patterns demonstrated to lower systemic inflammation and chronic disease risk across populations. In the context of IBD, these anti-inflammatory dietary approaches offer compelling benefits, including reduced disease activity, lower inflammatory markers, decreased reliance on corticosteroids, improved mucosal healing, and enhanced patient-reported outcomes such as quality of life and reduced fatigue (11). In this context, we conceptualize ‘anti-inflammatory dietary diversity' not merely as the consumption of varied foods, but as the strategic and synergistic intake of distinct bioactive compound classes—including soluble fibers, omega-3 fatty acids, and polyphenols—that engage non-overlapping immunomodulatory pathways to collectively reinforce mucosal homeostasis. This perspective examines key anti-inflammatory dietary approaches, their mechanisms, clinical evidence, limitations, and future directions in IBD.

## Fundamental principles linking diet to IBD pathophysiology

Diet exerts a profound influence on the intestinal ecosystem, serving as a primary source of substrates for microbial metabolism while simultaneously modulating host epithelial barrier function and immune homeostasis. Pro-inflammatory dietary patterns, characterized by excessive consumption of ultra-processed foods, refined carbohydrates, saturated fats, red and processed meats, and synthetic additives, profoundly perturb microbiota composition and function. These diets induce dysbiosis marked by reduced microbial diversity, diminished production of short-chain fatty acids (SCFAs), compromised tight junction integrity, and exaggerated Th17-driven inflammatory responses—hallmarks that mirror the core pathophysiological features of IBD (12). A salient example involves dietary emulsifiers, such as carboxymethylcellulose and polysorbate-80, which are prevalent in processed foods; these compounds disrupt the protective mucus layer overlying the intestinal epithelium, thereby facilitating bacterial adhesion to the mucosal surface and subsequent translocation into submucosal tissues (13, 14). Preclinical evidence (15) from animal models further substantiates this pro-inflammatory cascade, demonstrating that exposure to specific dietary-derived metabolites, such as secondary bile acids, intensifies colitis through activation of the NLRP3 inflammasome, culminating in enhanced release of interleukin-1β (IL-1β) and perpetuation of mucosal inflammation. In stark contrast, anti-inflammatory dietary patterns—abundant in whole foods, dietary fiber, polyphenols, omega-3 polyunsaturated fatty acids, and prebiotic compounds—actively mitigate these deleterious effects by fostering a resilient and beneficial microbial community while reinforcing host defensive mechanisms. Prebiotic fibers derived from fruits, vegetables, legumes, and whole grains selectively nourish SCFA-producing bacterial genera, including *Roseburia* and *Bifidobacterium*. The resultant SCFAs engage specific G-protein-coupled receptors on epithelial and immune cells, primarily GPR41 (also known as free fatty acid receptor 3, FFAR3) and GPR43 (FFAR2). Notably, butyrate also functions as a high-affinity ligand for GPR109A (hydroxycarboxylic acid receptor 2, HCAR2/niacin receptor 1), a receptor with a distinct signaling profile linked to inflammasome regulation and colonic regulatory T cell differentiation (16). While GPR41/43 mediate the broad metabolic and anti-inflammatory effects of acetate and propionate, GPR109A represents a specific butyrate-sensing mechanism that is particularly relevant to the effects described in Macia et al. (16). Similarly, polyphenols sourced from extra-virgin olive oil, berries, and other plant-rich foods confer potent antioxidant properties, attenuating oxidative stress and downregulating the production of key pro-inflammatory cytokines, such as tumor necrosis factor-α (TNF-α) and interleukin-6 (IL-6), via activation of the Nrf2 transcription factor pathway (17). Omega-3 fatty acids, predominantly from fatty fish and certain plant sources, favorably alter eicosanoid biosynthesis by competing with arachidonic acid pathways, shifting the balance toward the generation of anti-inflammatory and pro-resolving mediators, including resolvins and protectins, which facilitate the active resolution of inflammation and tissue repair (18). Emerging lines of investigation further illuminate intricate diet-microbiota-immune interactions, revealing that plant-predominant diets enrich mucinophilic species such as Akkermansia muciniphila, which has been shown in preclinical models to enhance mucosal barrier integrity (19). Collectively, these multifaceted mechanisms underscore the potential of targeted dietary interventions to fundamentally alter the trajectory of IBD when implemented early in the disease course, extending far beyond transient symptom amelioration to achieve meaningful modulation of inflammatory processes and promotion of long-term remission. Nonetheless, considerable inter-individual heterogeneity in treatment response persists, attributable to host genetic predispositions—such as NOD2 mutations that impair microbial pattern recognition—and variations in baseline microbiota composition and disease phenotype (20). Such variability underscores the imperative for personalized nutritional strategies tailored to individual profiles. Supporting this paradigm, longitudinal cohort studies have consistently demonstrated that sustained adherence to anti-inflammatory dietary patterns is associated with significant reductions in surrogate markers of inflammation, including fecal calprotectin levels, as well as improved endoscopic mucosal healing scores, thereby affirming a substantive preventive and therapeutic role for diet in IBD management (21, 22).

## Major anti-inflammatory dietary strategies in IBD

Several structured dietary interventions have been developed to target the inflammatory pathways in IBD, leveraging the principles of reducing pro-inflammatory triggers while promoting beneficial microbial and immune responses. These approaches vary in their composition, mechanisms, and levels of evidence, but collectively represent patient-centered adjuncts to conventional pharmacotherapy.

### Mediterranean diet

The Mediterranean diet (MD) is characterized by a high intake of fruits, vegetables, whole grains, legumes, nuts, extra-virgin olive oil, and fatty fish, coupled with moderate consumption of dairy and poultry, and minimal red or processed meats and refined sugars (23). Its potent anti-inflammatory effects are largely attributable to abundant polyphenols, monounsaturated fats, and omega-3 polyunsaturated fatty acids (24). Observational studies have consistently associated greater adherence to the MD with reduced disease activity scores and lower inflammatory markers in patients with IBD (25). In the context of active Crohn's disease, randomized trials employing MD-based or hybrid interventions have demonstrated significant improvements in clinical symptoms, quality of life, and nutritional status, often comparable to more restrictive diets. Mechanistically, the MD enhances the abundance of SCFA-producing bacteria, modulates adaptive immune responses through favorable T-cell polarization, and attenuates oxidative stress, thereby supporting mucosal healing and long-term remission maintenance (26).

### Specific carbohydrate diet

The Specific Carbohydrate Diet (SCD) is a strict elimination protocol that excludes complex carbohydrates, disaccharides, most grains, and certain starches, aiming to starve potentially pathogenic bacteria of fermentable substrates while permitting monosaccharides, proteins, and fats (27). In pediatric populations with Crohn's disease, early observational studies and small randomized trials have reported notable symptomatic relief, reductions in fecal calprotectin, and improvements in mucosal inflammation (28). Evidence in ulcerative colitis remains more limited, primarily derived from observational cohorts showing decreased calprotectin levels (29). However, the landscape of evidence for SCD shifted significantly with the publication of the landmark DINE-CD trial (Diet to INducE remission in Crohn's Disease) (11). This rigorous, randomized controlled trial compared the SCD directly to the Mediterranean diet (MD) in 191 adults with mild-to-moderate Crohn's disease. Crucially, the DINE-CD trial (11) found that SCD was not superior to the Mediterranean diet for achieving either symptomatic remission or reductions in fecal calprotectin. Both diets were associated with clinical improvement, but the MD offered advantages in terms of palatability and ease of adherence. Consequently, while SCD may remain a viable option for highly motivated patients (30), particularly in the pediatric setting, it is no longer considered a first-line dietary intervention for adult CD in light of the high-quality comparative evidence from DINE-CD.

### IBD-anti-inflammatory diet and groningen anti-inflammatory diet

The IBD-Anti-Inflammatory Diet (IBD-AID) builds upon the SCD framework by incorporating phased introduction of prebiotic and probiotic foods while rigorously avoiding individualized dietary triggers (31). Pilot investigations have revealed enhancements in microbial diversity, restoration of beneficial taxa, and clinical remission in a substantial proportion of patients (32). Similarly, the Groningen Anti-Inflammatory Diet (GrAID) emphasizes anti-inflammatory whole foods—including lean meats, certain dairy products, fruits, vegetables, and selected grains—while excluding refined sugars and processed items (33). It offers structured, evidence-informed recommendations tailored to IBD pathophysiology (34). Ongoing trials are exploring synergistic effects with micronutrient supplementation to further optimize microbiome modulation (35).

Additional promising strategies include the Crohn's Disease Exclusion Diet (CDED), which systematically eliminates processed foods, emulsifiers, and other additives, often combined with partial enteral nutrition to induce and sustain remission in both pediatric and adult Crohn's disease cohorts (36). Plant-based and semi-vegetarian diets, rich in antioxidants and fiber, have also demonstrated reductions in oxidative stress and lower relapse rates, particularly in maintaining remission through sustained anti-inflammatory effects (37).

## Clinical studies

The clinical evidence supporting anti-inflammatory dietary interventions in inflammatory bowel disease (IBD) has expanded considerably in recent years, with multiple randomized controlled trials (RCTs) demonstrating benefits in symptom relief, reduction of inflammatory markers, and achievement or maintenance of remission. Although study designs remain heterogeneous—varying in population (adult vs pediatric, Crohn's disease [CD] vs ulcerative colitis [UC]), intervention specifics, duration, and outcome measures—the collective data highlight the potential of structured dietary approaches to complement conventional pharmacological therapy. Limitations persist, including relatively small sample sizes, challenges with blinding and adherence monitoring, and insufficient powering for stringent endpoints such as endoscopic mucosal healing or steroid-free remission. Nevertheless, recent RCTs and prospective trials consistently report clinically meaningful improvements, particularly in symptom-oriented outcomes and quality of life.

Table 1 summarizes selected high-quality recent clinical studies (2024–2025) representing major anti-inflammatory dietary approaches. These trials illustrate sustained clinical benefits, including high rates of remission maintenance, reductions in fecal calprotectin (FC) and C-reactive protein (CRP), and improvements in disease activity scores. Emerging patterns suggest that diets emphasizing whole foods, reduced processed additives, Mediterranean principles, or adjunctive strategies (e.g., partial enteral nutrition) are safe, well-tolerated, and effective adjuncts.

**Table 1:** Summary of Key Clinical Studies on Anti-Inflammatory Diets in IBD.

Reference	Population	Intervention (vs Control)	Duration	Core outcomes measured	Key findings	Clinical implications
Marsh et al. (49)	Adults with IBD (mixed CD/UC)	IBD anti-inflammatory diet reducing additives (IBD-MAID; meals provided) vs general healthy eating advice	8 weeks	Disease activity (CDAI/HBI, SCCAI), symptoms, QoL, FC, CRP	High adherence (92%); significant within-group improvements in symptoms (*P* = 0.001), QoL (*P* = 0.004), FC (*P* = 0.007), and CD activity (*P* = 0.03) in IBD-MAID; reduced additive intake correlated with better markers	Reducing food additives may enhance anti-inflammatory effects and symptom control; supports personalized dietary modification
Erol Dogan et al. (50)	Mild-to-moderate active UC	Mediterranean diet (MD) alone vs MD + curcumin (1600 mg/day) vs MD + resveratrol (500 mg/day)	8 weeks	Disease activity (Truelove-Witts), inflammatory markers, QoL (SF-36, IBDQ)	All groups showed significant within-group reductions in disease activity and inflammation, and improved QoL (*P* < 0.05); no between-group differences except minor QoL sub-scores	MD is safe and effective for active UC; adjunctive nutraceuticals provide limited additional benefit
Narimani et al. (51)	Active mild-to-moderate UC	Combined Mediterranean + low-FODMAP diet + partial enteral nutrition vs regular diet	6 weeks	Disease activity index, QoL, hs-CRP, total antioxidant capacity	Significant decrease in disease activity (*P* = 0.043 between groups) and hs-CRP (*P* < 0.01 within intervention); improved QoL (*P* < 0.001)	Hybrid dietary approaches targeting inflammation and symptoms are highly effective and practical for UC management
Limketkai et al. (52)	Mild-to-moderate CD	Natural whole-food diet (healthful principles guidance) vs habitual diet	8 weeks	Clinical remission, FC change, Healthy Eating Index (adherence)	Higher remission in intervention (OR 1.41, *P* = 0.03); greater FC reduction with adherence (*P* = 0.047); no nutritional deficiencies	Emphasis on overall diet quality (“healthfulness”) drives clinical benefits; feasible and safe adjunct therapy
Preda et al. (26)	IBD (CD and UC) in remission	Anti-inflammatory diet (excluding red/processed meat, fried foods, fast food, etc.) vs regular diet	1 year	Maintenance of clinical remission, FC	Remission maintained: 95.2% vs 85.7% (*P* = 0.036); trend toward lower FC in intervention	Long-term anti-inflammatory dietary patterns support sustained remission and may reduce relapse risk

IBD, inflammatory bowel disease; CD, Crohn's disease; UC, ulcerative colitis; CDAI, Crohn's Disease Activity Index; HBI, Harvey-Bradshaw Index; SCCAI, Simple Clinical Colitis Activity Index; FC, fecal calprotectin; CRP, C-reactive protein; hs-CRP, high-sensitivity C-reactive protein; QoL, quality of life; IBDQ, Inflammatory Bowel Disease Questionnaire; OR, odds ratio.

Collectively, the recent RCTs summarized in Table 1 underscore a paradigm shift in dietary management of IBD—from empiric, highly restrictive elimination diets toward evidence-based, whole-food patterns that prioritize diet quality, sustainability, and patient acceptability. Across diverse populations and intervention designs, consistent signals emerge: structured reduction of ultra-processed foods and food additives, adherence to Mediterranean principles, and strategic incorporation of hybrid regimens confer statistically significant and clinically meaningful improvements in disease activity, inflammatory burden, and quality of life. A robust body of evidence, bolstered by recent systematic reviews and meta-analyses, now substantiates the efficacy of anti-inflammatory dietary interventions in inducing clinical remission, normalizing inflammatory biomarkers (e.g., fecal calprotectin [FC], C-reactive protein [CRP]), and promoting endoscopic mucosal healing (38, 39). Notably, the 2025 network meta-analysis by Wei et al. (38) provides the first comparative ranking of 15 dietary interventions, establishing that while the low-FODMAP diet—primarily a strategy for managing functional symptoms—demonstrates the greatest efficacy in reducing systemic inflammation (C-reactive protein) when combined with enteral nutrition, the Mediterranean diet combined with low-FODMAP and enteral nutrition optimizes patient-reported quality of life. These findings offer clinicians an evidence-based hierarchy to guide personalized dietary prescriptions that can simultaneously address inflammatory targets and symptom burden. However, methodological challenges persist across studies, including difficulties in blinding participants and investigators, inconsistent dietary adherence monitoring, and dropout rates approaching 30% in some trials.

## Challenges and future directions

Despite this robust mechanistic foundation and accumulating clinical efficacy data, several persistent and interconnected challenges impede the widespread translation of dietary therapy into routine IBD care. Foremost among these is the substantial inter-individual heterogeneity in treatment response, which arises from complex interactions among baseline microbiota composition, host genetic polymorphisms (e.g., NOD2 mutations impairing microbial sensing), disease phenotype, and concomitant pharmacotherapy (40). This variability precludes uniform efficacy and underscores the critical imperative for precision nutrition approaches. Methodologically, the field remains constrained by heterogeneous trial designs, relatively short follow-up durations (predominantly 6–8 weeks), inadequate blinding procedures inherent to dietary interventions, and inconsistent adherence monitoring—limitations that collectively downgrade the certainty of evidence per GRADE criteria and impede regulatory endorsement (41, 42). The recent publication of the European Crohn's and Colitis Organization (ECCO) Consensus on dietary management of IBD represents a landmark advance in addressing prior uncertainties by providing the first standardized, evidence-ranked clinical practice guidelines for dietary therapy in IBD (43). This consensus elevates diet from an ancillary consideration to a recognized therapeutic modality, offering clear, phenotype-specific recommendations—such as exclusive enteral nutrition (EEN) as first-line induction therapy in Crohn's disease, the Crohn's Disease Exclusion Diet plus partial enteral nutrition (CDED+PEN) as an effective alternative in pediatric and mild-to-moderate adult CD, and consideration of the Mediterranean diet as an adjunct for maintenance in ulcerative colitis—while strongly emphasizing universal access to IBD-experienced dietitians for personalized assessment, implementation, and monitoring. Nevertheless, the consensus openly acknowledges that the quality of evidence supporting many dietary interventions remains low or insufficient, which limits the strength of recommendations and continues to foster clinical hesitancy in fully integrating nutrition as a core therapeutic pillar (44).

Beyond methodological and biological challenges, profound socioeconomic and psychosocial barriers jeopardize the sustainability and equity of dietary interventions. Access to high-quality fresh fruits, vegetables, lean proteins, and anti-inflammatory fats is disproportionately constrained by food insecurity, geographic food deserts, and the higher relative cost of whole foods compared to ultra-processed alternatives. Simultaneously, patients with IBD frequently develop pathological food-related anxiety, avoidance behaviors, and disordered eating patterns—collectively termed “food-related anxiety”—which, if unaddressed by multidisciplinary care, paradoxically exacerbate malnutrition and diminish quality of life (45). These barriers mandate that dietary counseling extend beyond nutrient prescriptions to encompass culturally adapted, economically feasible, and psychologically informed implementation strategies.

Within this complex landscape, anti-inflammatory diets are optimally positioned as adjuncts to—rather than replacements for—pharmacological therapy, operationalized through shared decision-making and supervised by registered dietitians with IBD expertise to ensure nutritional adequacy, individualization, and sustained adherence (46). Tailoring dietary prescriptions to disease phase and predominant symptomatology offers a pragmatic framework: during active inflammation, Mediterranean-based or hybrid regimens (e.g., Mediterranean diet combined with partial enteral nutrition) prioritize direct inflammatory suppression; in quiescent disease complicated by irritable bowel syndrome-like symptoms, short-term, structured low-FODMAP diet under dietitian supervision addresses functional complaints without compromising inflammatory control (47); and for long-term remission maintenance, sustainable whole-food patterns emphasizing diet quality and anti-inflammatory diversity are favored.

Looking forward, the research agenda must advance decisively beyond feasibility pilots toward adequately powered, multi-center randomized controlled trials that mandate stringent, regulatory-grade endpoints—including steroid-free remission, endoscopic mucosal healing, and histologic remission—as co-primary outcomes (45). The integration of multi-omics technologies (shotgun metagenomics, metabolomics, proteomics, and host genotyping) within these trials is essential to deconstruct non-response, identify robust responder signatures, and enable pre-intervention patient stratification, thereby transitioning dietary therapy from empiricism to precision medicine. Concurrently, dedicated mechanistic investigations are urgently needed to elucidate the specific pathogenic effects of individual food additives (e.g., carboxymethylcellulose, polysorbate-80, titanium dioxide) and establish evidence-based thresholds for safe consumption (14). Promising ongoing initiatives, such as the VITA-GrAID trial (NCT04913467) (35), exemplify the next generation of research by evaluating combined dietary and micronutrient strategies in high-risk IBD cohorts, incorporating microbiome and metabolomic endpoints as mechanistic intermediates.

Ultimately, the emergence of personalized nutrition—guided by individual microbial, metabolic, and genetic profiles—holds transformative potential to resolve the paradox of inter-individual variability and unlock the full therapeutic efficacy of anti-inflammatory diets (48). Realizing this vision will require sustained interdisciplinary collaboration among gastroenterologists, dietitians, microbiologists, behavioral scientists, and health policy experts, alongside health systems-level innovations to ensure equitable access to medical nutrition therapy. Embedded within comprehensive, multidisciplinary IBD care models, structured anti-inflammatory dietary interventions are poised to evolve from an underutilized complementary strategy to an indispensable, evidence-based cornerstone of long-term disease modification.

## Conclusion

Anti-inflammatory dietary interventions represent a safe, patient-empowering adjunct in IBD management, capable of modulating microbiota-immune interactions to complement conventional pharmacotherapy. The therapeutic application of these diets is highly nuanced and must be stratified by disease phenotype (Crohn's disease vs. ulcerative colitis) and phase of illness (induction vs. maintenance of remission).

According to the 2025 European Crohn's and Colitis Organization (ECCO) consensus and recent network meta-analyses (38, 43), exclusive enteral nutrition (EEN) and the Crohn's Disease Exclusion Diet plus partial enteral nutrition (CDED+PEN) currently hold the highest level of evidence for induction of remission in luminal Crohn's disease. For maintenance of remission, particularly in ulcerative colitis, the Mediterranean diet demonstrates a favorable risk-benefit profile and is supported by consistent, albeit lower-grade, evidence. While emerging patterns like the IBD-Anti-Inflammatory Diet (IBD-AID) and low-additive diets show promise in pilot studies, they require validation in larger comparative RCTs. Embedding structured nutritional strategies within multidisciplinary care models, guided by IBD-specialist dietitians, has the potential to substantially improve long-term outcomes.

## Data Availability

The original contributions presented in the study are included in the article/supplementary material, further inquiries can be directed to the corresponding authors.
